# Isocaloric Diets with Different Protein-Carbohydrate Ratios: The Effect on Sleep, Melatonin Secretion and Subsequent Nutritional Response in Healthy Young Men

**DOI:** 10.3390/nu14245299

**Published:** 2022-12-13

**Authors:** Oussama Saidi, Emmanuelle Rochette, Giovanna Del Sordo, Paul Peyrel, Jérôme Salles, Eric Doré, Etienne Merlin, Stéphane Walrand, Pascale Duché

**Affiliations:** 1 Laboratory Impact of Physical Activity on Health (IAPS), Toulon University, F-83041 Toulon, France; 2 Laboratory of Adaptations to Exercise under Physiological and Pathological Conditions (AME2P), Clermont Auvergne University, F-63000 Clermont-Ferrand, France; 3 Center for Research in Human Nutrition Auvergne, F-63000 Clermont-Ferrand, France; 4 Department of Pediatrics, Clermont-Ferrand University Hospital, F-63000 Clermont-Ferrand, France; 5 INSERM, CIC 1405, CRECHE Unit, Clermont Auvergne University, F-63000 Clermont-Ferrand, France; 6 Department of Kinesiology, Laval University, Quebec, QC G1V 0A6, Canada; 7 Quebec Heart and Lung Institute, Laval University, Quebec, QC G1V 4G5, Canada; 8 Human Nutrition Unit, INRAE, Auvergne Human Nutrition Research Center, Clermont Auvergne University, F-63000 Clermont-Ferrand, France

**Keywords:** macronutrient, tryptophan, REM sleep, cortical arousal

## Abstract

This study aimed to determine the short-term effect of two isocaloric diets differing in the ratio of protein–carbohydrate on melatonin levels, sleep, and subsequent dietary intake and physical activity in healthy young men. Twenty-four healthy men took part in a crossover design including two sessions of three days on isocaloric diets whether high-protein, low-carbohydrate (HPLC) or low-protein, high-carbohydrate (LPHC) followed by 24-h free living assessments. Sleep was measured by ambulatory polysomnography pre-post-intervention. Melatonin levels were assessed on the third night of each session on eight-point salivary sampling. Physical activity was monitored by accelerometry. On day 4, participants reported their 24-h ad-libitum dietary intake. LPHC resulted in better sleep quality and increased secretion of melatonin compared to HPLC. A significant difference was noted in sleep efficiency (*p* < 0.05) between the two sessions. This was mainly explained by a difference in sleep onset latency (*p* < 0.01) which was decreased during LPHC (PRE: 15.8 ± 7.8 min, POST: 11.4 ± 4.5 min, *p* < 0.001). Differences were also noted in sleep staging including time spent on REM (*p* < 0.05) and N1 (*p* < 0.05). More importantly, REM latency (PRE: 97.2 ± 19.9 min, POST 112.0 ± 20.7 min, *p* < 0.001) and cortical arousals (PRE: 7.2 ± 3.9 event/h, POST 8.5 ± 3.3 event/h) increased in response to HPLC diet but not LPHC. On day 4, 24-h *ad-libitum* energy intake was higher following HPLC compared to LPHC (+64 kcal, *p* < 0.05) and explained by increased snacking behavior (*p* < 0.01) especially from carbohydrates (*p* < 0.05). Increased carbohydrates intake was associated with increased cortical arousals.

## 1. Introduction

Sleep and circadian rhythms play a critical role in the proper functioning of several physiological processes, including metabolic regulation, energy balance, and weight control [1]. Although the effect on energy expenditure remains unclear, growing evidence points to the key role of sleep duration and quality on subsequent nutritional response. Inadequate sleep duration and quality lead to increased food intake due to altered hunger and satiety, as well as pronounced cravings for energy-dense foods [2,3]. On the other hand, it appears that the nutrients consumed may also exert ongoing feedback on the body clock and sleep physiology [4].

Given its important public health implications, the effect of nutrition on sleep has received much attention during the last decades [5,6]. However, dietary patterns include several parameters, making it difficult to draw definitive conclusions [7]. For instance, when focusing on the link between protein or carbohydrates and sleep, the overall picture is mixed, to say the least [4,8]. More specifically, the existing evidence, does not allow to draw a consensus about the potential effects of protein or carbohydrates on sleep [9]. A cross-sectional study indicates that while low protein intake (<16% of energy intake) is associated with greater sleep latency, high protein intake (>19% of energy intake) was associated with sleep fragmentation [10]. An observational study based on polysomnography sleep measurement in 50 healthy adults found that high protein, low carbohydrate consumption (HPLC) was associated with an increased proportion of rapid eye movement (REM) sleep [11]. Lindseth et al., (2013) reported a reduction of wake after sleep onset following the consumption of a high protein diet [12]. However, they reported detrimental effects on sleep continuity in another study [13]. Nevertheless, it was also suggested that high protein intake under energy restriction helps maintain and enhance sleep quality [14]. Regarding carbohydrates and sleep, Tan et al., (2015) found that individuals with sleep disorders had a lower proportion of carbohydrates compared to healthy sleepers [15]. Several studies reported positive effects on REM sleep with a reduction of wake time and sleep onset latency. However, non-rapid eye movement (NREM) was reduced [12,13,16,17,18,19]. Several methodological deficiencies make the comparability of these results very difficult. First, we found that the caloric content was not systematically matched in previous studies. If not, the comparison often referred to a mixed control diet accompanied by an adjustment in fat intake that can potentially interfere with the results. More surprisingly, we also noticed a complete omission of the potential interaction between macronutrients at the physiological level. However, these interactions have been put forward by the serotoninergic hypothesis [20] and this may explain, at least in part, the discrepancy in the current literature.

Taking into account all of these inherent challenges, in this study, we sought to determine the short-term effect of two isocaloric diets, either a high-protein, low-carbohydrate diet (HPLC) or a low-protein, high-carbohydrate diet (LPHC), on objectively measured sleep and melatonin secretion. Previously, Boelsma et al., (2010) found that the HPLC diet induced an increase in postprandial alertness compared to the LPHC diet [21]. Therefore, it was hypothesized that the HPLC diet would increase arousal and impair sleep quality compared to the LPHC diet according to the serotoninergic hypothesis. Given that sleep is a key factor affecting the regulation of energy balance, we also explored the effect of both diets (HPLC vs. LPHC) on subsequent 24-h free-living ad-libitum dietary intake and physical activity.

## 2. Materials and Methods

### 2.1. Participants

Participants were recruited from college students through university e-mail between October 2018 and June 2020. A telephone conversation was scheduled with interested individuals to determine eligibility, conduct screening, and introduce the experimental protocol of the study. To be included, participants had to be young men (19–25 years) in good health, within the “normal” range for body mass index (20–25 kg/m^2^), free of medication that may interfere with the main outcomes (e.g., corticosteroids, anti-depressants, anxiolytics, non-steroidal anti-inflammatory drugs, etc.), and not following any special diet. They ought not to have a risk for sleep apnea as assessed by the “Berlin” questionnaire [22] nor diagnosed psychiatric, circadian rhythm, and sleep disorders. Twenty-four participants completed the study successfully and were retained in the analysis.

All study procedures were approved by a local ethical authority (Comité d’Éthique pour la Recherche en Sciences et Techniques des Activités Physiques et Sportives CERSTAPS, clearance certificate no. [2018-03-10-26]). The experimental design was in agreement with the policy statement regarding the use of human subjects by the Declaration of Helsinki. Written informed consent was obtained from every participant prior to the study launch. Participants were aware that we were interested in studying the effect of diet on sleep but were unaware of any potential effect of the intervention on subsequent dietary intake and physical activity.

### 2.2. Study Procedure

Before the experimental sessions, the participants take part in a one-week run-in period. They underwent anthropometrical and body composition assessments. They also completed questionnaires about their chronotype: The Horne–Östberg Morningness-Eveningness Questionnaire (MEQ) [23], subjective sleep quality: The Pittsburgh Sleep Quality Index (PSQI) [24], and habitual physical activity: The International Physical Activity Questionnaire short form (IPAQ-SF) [25]. Participants were also briefed on the details of all study measurements, notably the in-home salivary melatonin collection protocol. They were properly trained to operate the ambulatory polysomnography device (PSG) (Sleep Profiler PSG2, Advanced Brain Monitoring, Carlsbad, CA, USA) and underwent at least one habituation night to become familiar sleeping with the device. The sleep schedule was stabilized from 23:00 to 07:00 in order to reduce intra-individual variability, and compliance was verified using a sleep diary and accelerometers (ActiGraph, Pensacola, FL, USA). They were also invited to an appointment with a dietitian who explained the nutritional plan of each session, the methods of preparation of meals, and provided a kitchen scale. Participants were asked to precisely adhere to the instructions provided by the dietitian and to report any failure in complying with the experimental session diets.

**Figure 1:**
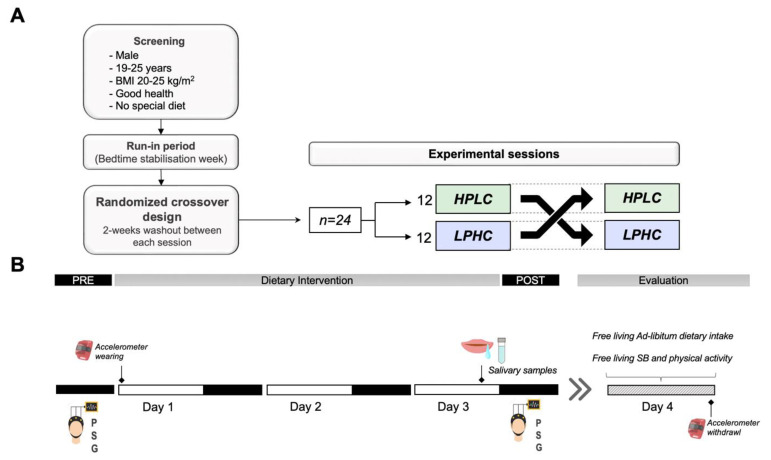
Study overview. (**A**) A randomized, crossover study design including two sessions on isocaloric diets whether HPLC or LPHC. (**B**) Each session comprised three days of intervention during which sleep was measured by polysomnography. Salivary samples were collected on the third day of each session from melatonin assessment. The intervention was followed by 24-h free living evaluation of physical activity, and ad-libitum dietary intake; SB: sedentary behavior.

After this run-in period, participants take part in a randomized crossover design including two sessions with three days on isocaloric diets whether HPLC (Protein/Carbohydrates ratio = 0.44) or LPHC (Protein/Carbohydrates ratio = 0.20) separated by a two-week washout (Figure 1). They were asked to refrain from any vigorous physical activity (e.g., training) during the experimental days of the protocol and to maintain the same routine activities between sessions. Time spent in sedentary behaviors and activity intensities were monitored by accelerometry (Table S3). Evening use of electronic devices was prohibited during the entire study. In-home polysomnography recordings (Sleep Profiler-PSG2™, Advanced Brain Monitoring Inc.) were used to measure sleep the night of day 0 of each session (PRE-intervention), and the night of day 3 (POST-intervention). Melatonin levels were assessed on the third night of each session. On day 4, participants underwent 24-h free living evaluation of physical activity, and ad-libitum dietary intake.

### 2.3. Nutritional Intervention

Two nutritional plans were designed by a registered dietician (Table S1). Both diets (HPLC and LPHC) were not energy-restrictive. Energy intake was adapted to each participant’s energy requirement according to measured resting metabolic rate and level of physical activity. They were iso-energetic and contained the same meals. However, HPLC diet contains 2 g/kg of protein, and 4.45 g/kg of carbohydrates (CHO), whereas LPHC diet contains 1.1 g /kg of protein, and 5.35 g/kg of CHO resulting in a different protein/CHO ratio (HPLC = 0.44 versus LPHC = 0.20). The amount of protein-rich foods (e.g., chicken, turkey) was increased while the amount of carbohydrate-rich foods (e.g., bread, pasta) was decreased in HPLC and vice-versa for the LPHC diet. Fat intake was fixed in both diets. All meals were scheduled similarly between sessions and days: 7:30 (breakfast), 12:30 (lunch), and 19:30 (dinner). Caffeinated and alcoholic beverages consumption were prohibited during the experimental protocol. The nutritional plans of the third day of each session did not include food rich in melatonin (e.g., chocolate, banana, tomatoes, nuts). Details of food items included in the nutritional plans are given in Table S2.

### 2.4. Measurements

#### 2.4.1. Anthropometric Characteristics and Body Composition

Height was measured in a barefoot standing position, using a calibrated stadiometer. Body mass (BM), fat mass (FM), and fat-free mass (FFM) were assessed using a TANITA TBF-300 impedance meter (TANITA Corporation, Tokyo, Japan). Body mass index (BMI) was calculated as BM divided by height squared in (kg/m^2^).

#### 2.4.2. Physical Activity and Sedentary Time

Physical activity and sedentary time were monitored during the whole experimental sessions period using GT3X tri-axial accelerometers (ActiGraph, Pensacola, FL, USA). Participants were instructed to wear the accelerometer on the right hip, except when water contact is possible (showering, bathing). Accelerometers were initialized for data collection at 30 Hz using ActiLife software version 6.13.4 (ActiGraph, Pensacola, FL, USA). Data were reintegrated into 60-s epochs and then classified into intensity levels using the following cut-points: Moderate-to-vigorous physical activity (MVPA) ≥ 1952 counts per minute (CPM) and sedentary time < 100 CPM [26].

#### 2.4.3. Melatonin Profile/Phase Angles

Salivary samples were collected in the participant’s habitual sleep environment on the evening of day 0 and day 3 of each session. According to Pullman et al. (2012) participants were asked to collect eight saliva samples in dim light conditions to ensure documenting the increase of melatonin [27]. They started sampling (6, 5, 4, 3, 2, and 1 h) before bedtime. Saliva was then collected again at bedtime and participants were asked to set the alarm and wake to take a final sample one hour after bedtime. Participants were asked to allow saliva to accumulate in their mouth and then drool in the collector tube Salivette^®^ (Sarstedet, Nümbrecht, Germany). The saliva collection tubes were coded according to the collection time (6, 5, 4, 3, 2, 1 h before bedtime, bedtime, and 1 h after bedtime). After each hourly collection, the tube was placed in a labelled box at 4 °C. The next morning, participants bring the box containing the samples to the investigators. The samples were centrifuged at 5000× *g* for 15 min at 4 °C and then stored at −80 °C until analysis.

The recommended amount of saliva was 225 μL for the correct amount provided for the test, the amount to be tested for each saliva sample was 100 μL. Each saliva sample was analyzed separately, with the average concentration of melatonin indicated. The saliva test has a melatonin sensitivity detection as low as 1.37 μg / mL. Any sample containing less than 100 μL was reported as an insufficient quantity. Melatonin levels were assessed by immunoassay using the salivary kit from Salimetrics (Salimetrics, LLC, Carlsbad, CA, USA), according to the recommendations provided by the manufacturers, (https://salimetrics.com/wp-content/uploads/2018/03/melatonin-saliva-elisakit.pdf, accessed on 1 September 2018).

#### 2.4.4. Sleep

Sleep Profiler-PSG2™ (Advanced Brain Monitoring, Carlsbad, CA, USA) which is an ambulatory sleep device approved by the Food and Drugs Administration (FDA) was used to measure sleep. This system is reproducible and validated against golden standard in laboratory polysomnography among adults [28,29,30]. It provides access to thirteen channels: electroencephalography, electro-oculography, and electromyography from frontpolar sites, airflow through a nasal cannula and pressure transducer; head movement and position by actigraphy; snoring with an acoustic microphone; pulse from the forehead and finger; wireless wrist oximetry; and thorax and abdomen effort by respiratory induced plethysmography. The participants were accustomed to wearing and operating the device after a demonstration visit held in the laboratory. Thereafter, objective sleep assessment was based on night recordings (following day 0 and day 3). After each session, participants returned the device to the investigator who extracted the records through the Sleep Profiler portal. Automated algorithms were applied to the signals. Auto-staging was performed based on the ratios of the power spectral densities and auto-detection of cortical and microarousals, sleep spindles, and ocular activity [29]. After the studies were processed, an experimented sleep expert reviews the recordings in order to confirm the accuracy of the auto-sleep staging. This tool provides access to total sleep time (TST), sleep latency (SOL), wake up after sleep (WASO), sleep efficiency (SE), awakenings of more than 30 s, awakenings of more than 90 s, arousal index, as well as sleep architecture according to the American Academy of Sleep Medicine (AASM) recommendations [31].

#### 2.4.5. Ad-Libitum Dietary Intake

On day 4, participants were instructed to consume foods “ad-libitum” (until satisfaction). They kept a nutritional log and were instructed to weigh and record all foods consumed throughout the day. After that, energy intake, macronutrients, and micronutrients composition were calculated using a professional computerized nutrient analysis program (Bilnut 4.0 SCDA Nutrisoft software) and Ciqual tables (year-2020 version).

### 2.5. Statistical Analysis

R Studio (version 4.0.5, RCore Team, 2021) and Prism 9 (GraphPad, San Diego, CA, USA) were used to perform statistical analysis and graphing. Data were expressed as mean ± standard deviation (SD) unless otherwise specified. Statistical inferences were drawn at 0.05 level of significance.

Manipulation check was performed (Wilcoxon tests for paired sample) on accelerometry data (Table S3) and PRE-intervention sleep outcomes (Table S4) to ensure that level of physical activity was not different between the two sessions and that the washout period was effective taking participants to baseline sleep.

For salivary melatonin secretion, eight measures were performed for each session. Each time point was compared between LPHC and HPLC using Wilcoxon tests for paired samples. The area under the curve (AUC) was computed for the eight measures of melatonin using the trapezoid method and compared between LPHC and HPLC using a Wilcoxon test for paired data. Deltas were computed between PRE and POST for HPLC and LPHC and compared to assess sleep variation following HPLC vs. LPHC. Furthermore, one-way ANCOVAs were computed on the delta between PRE and POST-test measures, with sessions as fixed factors and PRE measures as covariate. Wilcoxon tests and ANCOVAs results yielded similar results for all sleep parameters.

Comparison of dietary intake and physical activity on day 4 were computed using Wilcoxon tests for paired sample. Effect sizes’ r for Wilcoxon test were computed and can be interpreted as follows: 0.1–0.3 small effect, 0.3–0.5 medium effect, >= 0.5 large effect. Pearson’s correlations were performed to examine the relationships between Δ dietary intake outcomes on day 4 and Δ sleep outcomes between the two sessions on the previous night.

## 3. Results

### 3.1. Participants

Table 1 presents descriptive characteristics of participants. Briefly, participants were all normal weighted. Participants MEQ_score_ was on average 49.10 ± 10.30, according to which participants circadian phenotypes distribution was as follows: Morning-type: n = 5, Intermediate-type: n = 13, and Evening-type: n = 6. The mean PSQI score of the subjects was 4.79 ± 1.89. Participants level of sleepiness was within the normal range of daytime sleepiness in healthy adults, as measured by the Epworth Sleepiness Scale (ESS) score. Finally, the mean RMR of the participants was 1813 ± 135 kcal·day^−1^.

### 3.2. Melatonin Secretion

**Figure 2:**
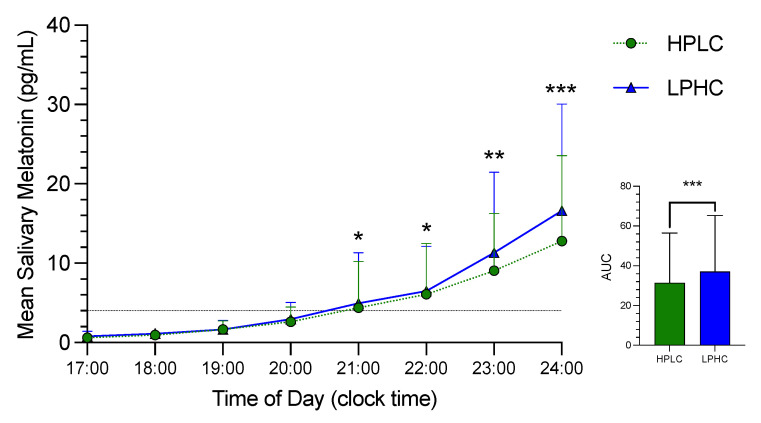
The time courses of salivary melatonin secretion and area under the curve (AUC) in HPLC vs. LPHC diets. HPLC: high-protein, low-carbohydrate; LPHC: low-protein, high carbohydrate. *: significant difference (HPLC vs. LPHC) with *p* < 0.05. **: significant difference (HPLC vs. LPHC) with *p* < 0.01. ***: significant difference (HPLC vs. LPHC) with *p* < 0.001.

Melatonin secretion from the eight saliva samples collected under dim-light conditions during the third night of HPLC and LPHC is shown in Figure 2. The first four saliva samples show no difference between the two sessions (HPLC vs LPHC). However, there is a small significant difference in melatonin secretion that appears starting at 21:00 (2 h before sleep time). The magnitude of the difference continued increasing until 1 h after sleep time (21:00: *p* < 0.05, ES = 0.09; 22:00: *p* < 0.05, ES = 0.11; 23:00: *p* < 0.01, ES = 0.13; 24:00: *p* < 0.001, ES = 0.21). Difference in melatonin secretion between the two sessions was achieved right after dim-light melatonin onset (DLMO). However, no circadian shift was detected (HPLC: 21:28 ± 1:24 vs. LPHC 21:22 ± 1:21, *p* = ns). Salivary melatonin AUC was higher during LPHC compared to HPLC (*p* < 0.001, ES = 0.15).

### 3.3. Sleep

The change in sleep parameters from night 0 (PRE) to night 3 (POST) following HPLC diet consumption shows no significant difference. However, SE tended to decrease (PRE: 88.7 ± 3.6%; POST: 87.8 ± 3.5, *p* = 0.057, [small effect]). This was marked by a tendency to increase in WASO (PRE: 38.5 ± 13.8 min; POST: 42.4 ± 12.4, *p* = 0.055, [small effect]. The change in sleep parameters from night 0 (PRE) to night 3 (POST) following LPHC diet consumption shows a decrease in SOL (PRE: 15.8 ± 7.8 min; POST: 11.4 ± 4.5 min, *p* < 0.001, [moderate effect] as well as proportion and absolute time spent on N1 stage [large effect]. The comparison of sleep change in HPLC compared to LPHC showed differences in SE (*p* < 0.05, [moderate effect]) and SOL (*p* < 0.01, [moderate effect]). Moreover, there was a difference in sleep staging variation including a decrease in N1 stage proportion (*p* < 0.05, [moderate effect]) and absolute time (*p* < 0.05, [moderate effect]) as well as REM sleep absolute time (*p* < 0.05, [moderate effect]) but not proportion in HPLC vs. LPHC (Table 2).

**Figure 3:**
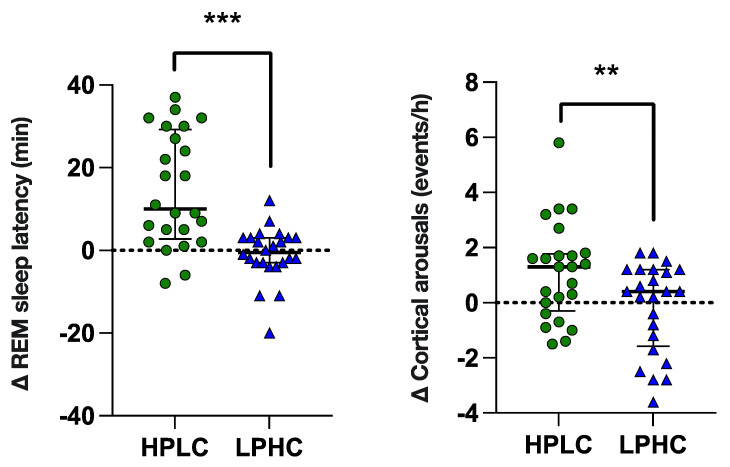
REM latency and arousals variation in response to HPLC vs. LPHC diets. Data are shown as median ± interquartile range with scatter-plots representing individuals’ data. *p* value: ** *p* < 0.01; *** *p* < 0.001.

As shown in Figure 3, there were also marked differences in REM sleep latency and cortical arousals variation (*p* < 0.001, [large effect]; *p* < 0.01, [moderate effect]), respectively) in response to HPLC vs. LPHC diets. As shown in Figure S1, the change in REM sleep latency and cortical arousals from night 0 (PRE) to night 3 (POST) was significant only following HPLC diet consumption.

### 3.4. Day 4 Free Living Ad-Libitum Dietary Intake

Free living 24-h ad-libitum energy and macronutrients intake during day 4 in response to HPLC vs. LPHC are reported in Table 3. Although the magnitude of the effect was small, there was an increase on total energy intake following HPLC vs. LPHC (+63 ± 104 kcal, *p* < 0.05) especially from total CHO intake (*p* < 0.007). This was mainly due to an increase in energy intake (EI) and CHO intake outside of meals (both snacking EI and CHO were increased, *p* < 0.01 and *p* < 0.05, respectively).

When analyzing the associations between changes in dietary intake outcomes during day 4 between HPLC and LPHC with that of sleep outcomes changes, a positive correlation between Δ cortical arousals and Δ CHO was found (r = 0.523, *p* < 0.01) (Figure S2).

### 3.5. Day 4 Free Living Time Spent on Sedentary Behaviors and Physical Activity

No differences were detected in free living sedentary behaviors and physical activity during day 4 between HPLC and LPHC (Table 4).

## 4. Discussion

The proportion of macronutrients and more specifically the protein-carbohydrate ratio are involved in sleep regulation. We show here a better sleep quality after three days of the LPHC diet compared to the HPLC diet (protein-carbohydrate ratio: 0.20 vs. 0.44, respectively) under isocaloric intake. The low protein–carbohydrate ratio diet (LPHC) reduced sleep latency and N1 sleep stage, whereas the high-ratio diet (HPLC) tended to increase wakefulness after sleep onset. Variation in sleep outcomes in response to the two diets revealed differences in sleep efficiency, sleep latency, and staging (REM and N1 sleep stages). More importantly, there were marked differences in REM sleep latency and cortical arousals that increased with the high ratio diet (HPLC) but not with the low ratio diet (LPHC).

One of the major plausible explanations for the effect of diet on the central nervous system and subsequent sleep was proposed based on the serotonergic hypothesis [6]. Serotonin, also called 5-hydroxytryptamine (5-HT), is converted to melatonin through the serotonin pathway and both molecules play a paramount role in sleep physiology [32]. Furthermore, 5-HT cannot cross the blood-brain barrier (BBB). Therefore, its synthesis in the brain is exclusively achieved by the serotonergic neurons [33]. Tryptophan (Trp), the precursor of 5-HT biosynthesis, is an essential amino acid (i.e., it cannot be synthesized by the body and must be provided exclusively via dietary proteins). Following the breakdown of proteins during digestion, Trp is delivered to the bloodstream in two forms: most of it is bound to serum albumin, while the rest remains in free-form, 1–2% being used for 5-HT synthesis [34]. Only the free Trp fraction can cross the BBB notably through an active transporter, L-type amino acid transporter 1 (LAT-1), particularly present on the membrane of endothelial cells. However, Trp must compete with large neutral amino acids (LNAAs) when crossing the BBB [35]. Although Trp/ LNAAs ratio was not measured in the current study, it was assumed that macronutrients intake could modulate the uptake of Trp by the BBB and consequently affect melatonin synthesis. In fact, Trp is less abundant than LNAAs. Therefore, ingestion of high protein intake generally decreases Trp/LNAAs ratios in the blood which is hypothesized to decrease the uptake of Trp into the brain. Quite the contrary, carbohydrate promotes Trp travel to the brain through the stimulation of insulin release [36]. Insulin potentiates the uptake of LNAAs by the muscle and leads to higher Trp/LNAAs ratio [36,37]. The observed sleep variation under the high protein–carbohydrate ratio diet reminds early studies experimentally inducing rapid Trp depletion. Although sleep variation was more modest in the current study, our results were consistent with early findings. Voderholzer et al., (1998) reported an alteration of sleep continuity with an increased number of wakening after sleep onset, wake percentage, lighter sleep stages (increased time spent on N1 stage) as well as a delay in REM sleep manifestation (corrected REM sleep latency) and an increase in the total number of rapid eye movements during REM sleep after two days of low protein diet followed by the administration of amino acid mixture either without tryptophan or containing 2.3 g of tryptophan (placebo control) [38]. Bhatti et al., (1998) reported an increase in sleep onset latency and wake after sleep onset as well as a reduction in time spent on REM sleep subsequently to the consumption of (25% and 100%) tryptophan-free amino acid drink [39]. Arnulf et al., (2002) tested a mid-morning Trp depletion challenge and also found an increase in arousal index and REM sleep latency concomitant with an approximately 77% decrease in serum Trp levels [40]. These results were explained by a reduction in 5-HT release from neurons [41]. Even though serum Trp was not measured in the current study, we found here lower melatonin secretion following the high protein–carbohydrate ratio diet (HPLC) compared to the low ratio one (LPHC). This may reflect a decrease in Trp following HPLC as already shown by Wurtman et al., (2003) [42]. In the latter study a median difference of 54% was obtained after a carbohydrate-rich versus (+10% after 240 min) a protein-rich meal (−35% after 240 min) on plasma tryptophan-LNAA ratios. Moreover, a clinically significant rise in insulin was noted with the carbohydrate-rich meal which was not the case for the protein-rich meal.

Although several studies documented that experimental protocols of Trp depletion alters sleep [41], to the best of our knowledge, it has not yet been demonstrated that a HPLC in comparison to LPHC diet maintained for three consecutive days can reflect similar effects on sleep. An early study by Lacey et al. (1977) tested the immediate effect of injection of intravenous amino acids or glucose on sleep. They noticed that REM sleep was decreased with amino acids and increased with glucose [43]. Kwan et al., (1986) found that low-carbohydrate isoenergetic diet consumption for one week induced a delay in REM sleep manifestation [18]. Unfortunately, this study only reported sleep staging among a limited number of participants (six women). Furthermore, although the low protein to carbohydrates ratio was similar in this study to that of HPLC, the control diet showed a higher ratio compared to LPHC. Moreover, there was a notable adjustment of fat intake between the two-test diets which was not the case in our study. The work provided by Afaghi et al., (2008) on the Atkin’s diet effect on sleep showed a reduction in REM sleep accompanied by an increase in slow-wave sleep (SWS) during both the acute and ketosis state [16]. However, the Atkin’s diet is characterized by important increase in fat intake (61% of total EI). It is worth noting in this case that fat intake has a direct impact on post-prandial free fatty acids concentrations in the bloodstream. Free fatty acids are bounded by albumin and could, in turn, modulate the free Trp fraction. This could eventually explain the difference in the obtained response. It is true that some studies reported positive effects of high protein diets for weight loss on sleep [14]. By contrast, most of them were realized among overweight or patients with obesity under energy restriction. Zhou et al., (2016) indicate that an intake of 1.5 g/kg/day of protein concomitant with an energy restriction preserved sleep quality better than the intake of 0.8 g/kg/day. However, this study was limited by the subjective nature of sleep outcomes. More importantly, a previous study underlined that energy restriction altered sleep [44]. Thus, the effect of high protein intake on sleep may differ under altered energy homeostasis state.

Another important finding of this study was the increase in cortical arousals in HPLC vs. LPHC and the association of cortical arousals variation with next day CHO consumption. Previous studies showed that cortical arousals are stimulated by orexin system activity mainly through the aminergic nuclei [45] which permit the control of energy homeostasis. It was shown that both glucose and 5-HT inhibits orexin neurons [46,47,48]. Thus, HPLC may have resulted in an increase in cortical arousals through an activation of the orexin system due to low glucose and 5-HT levels which is supposed to increase food foraging behaviors [49]. Multiple studies showed that the activation of the orexin system play a key role in food rewards processing [49,50]. This was supported by the association of orexin system activity and opioid-induced palatable food intake in animal models studies [51].

This study overcame several methodological weaknesses reported in the literature. In particular, maintaining the same energy and fat intake while comparing HPLC to LPHC diets and fixing time spent in bed during the whole study. However, our results need to be endorsed by studies measuring plasma levels of Trp/LNAA, 5-HT, and Orexin neuropeptides. This is a major limitation that prevented us from giving a quantitative analysis associating protein–carbohydrate ratio effects on Trp/LNAA and sleep outcomes. In a recent review, Benton et al. (2022) were skeptical of the serotoninergic hypothesis and the ability of macronutrients manipulation in modulating Trp uptake by the brain. Instead, they proposed that sleep variation would be more attributable to the influence of carbohydrates on blood glucose based on the theory of sleep and energy homeostasis. In this case, they suggested that glucose signaling permits the regulation of energy balance and sleep by influencing orexin system activity. However, future human studies are needed to determine the optimal threshold of protein/carbohydrate ratio for better sleep and draw a clearer and more detailed picture on the effect of both mechanisms (serotonergic or blood glucose) on sleep. Moreover, the sample size in the current study might have underpowered the obtained significance, especially regarding next day free-living dietary intake, which could have been also affected by external factors such as social or food availability. Thus, future studies with a larger sample size are needed to evaluate the effect of sleep variation following the consumption of a particular diet on subsequent dietary intake using in-laboratory standardized ad-libitum meals accompanied by the evaluation of appetite sensations and the satiety quotient. These studies would be of major interest for the development of sleep friendly strategies for weight control or loss. We also emphasize that this study only included healthy young participants, potentially limiting the interpretation of our findings to other populations. Thus, next studies should also include different populations (participants with obesity, metabolic syndrome, etc.) with inherent sleep problems. Finally, it would also be interesting to include women and determine a potential sex effect on this response.

## 5. Conclusions

Diets with different protein–carbohydrate ratios may affect subsequent sleep. Our results show a better sleep quality after three days of the LPHC diet compared to the HPLC diet (protein–carbohydrate ratio: 0.20 vs. 0.44, respectively) under isocaloric intake. Differences in sleep efficiency, sleep latency, and staging (REM and N1 sleep stages) as well as marked differences in REM sleep latency and cortical arousals were detected. These effects could be explained by reduced melatonin secretion. However, low protein/CHO ratio was associated with improved sleep quality and increased melatonin secretion. Other potential contributor factors such as plasma levels of Trp/LNAA, 5-HT, and Orexin neuropeptides should be addressed in future human studies with a larger sample size in order to confirm these results and draw a clearer picture of the mechanistic pathways explaining this effect.

## Figures and Tables

**Table 1:** Characteristics of study participants.

	Mean (SD)	95% CI
Age (year)	22.80 (1.90)	(22–23)
Height (cm)	181 (7.36)	(178–184)
Weight (kg)	73.30 (7.30)	(70.21–76.38)
BMI (kg·m^−2^)	22.30 (1.54)	(21.68–22.98)
FM (%)	11.70 (3.64)	(10.21–13.28)
FFM (kg)	64.50 (5.17)	(62.34–66.71)
Circadian typology (MEQ_score_)	49.10 (10.30)	(44.79–53.46)
Sleep quality (PSQIscore)	4.79 (1.89)	(3.99–5.59)
Sleepiness (ESS_score_)	5.96 (3.08)	(4.65–7.26)
Sleep stabilization week sleep onset (time)	22:39 (00:13)	(1353–1364)
Sleep stabilization week total time in bed (h:min)	8:04 (00:15)	(476–490)
RMR (kcal·day^−1^)	1813 (135)	(1756–1870)

BMI: body mass index; CI: confidence interval; ESS: epworth Sleepiness Scale; FFM: fat free mass; FM: fat mass; MEQ: Horne–Östberg morningness-eveningness questionnaire; PSQI: Pittsburgh sleep quality index; RMR: resting metabolic rate; SD: standard deviation.

**Table 2:** Effect of HPLC vs. LPHC diets on sleep quality and staging.

	HPLC (n = 24)			LPHC (n = 24)			HPLC vs. LPHC	
	PRE Mean (SD)	POST Mean (SD)	*p*-Value (ES) of Differences	PRE Mean (SD)	POST Mean (SD)	*p*-Value (ES) of Differences	Delta HPLC-LPHC (SD)	*p*-Value (ES) of Differences
TST (min)	423 (24.00)	419 (25.30)	0.126 (0.11)	426 (24.60)	426 (24.40)	0.987 (0.02)	−4.50 (14.84)	0.290 (0.17)
REM (min)	108 (21.60)	102 (27.50)	0.119 (0.10)	109 (22.10)	112 (17.00)	0.145 (0.10)	−9.10 (23.82)	0.039 (0.32)
REM (%)	25.60 (4.94)	24.50 (6.44)	0.381 (0.08)	25.70 (5.43)	26.40 (4.16)	0.186 (0.10)	−3.00 (7.77)	0.053 (0.37)
N1 (min)	30.3 (5.33)	29.90 (10.20)	0.617 (0.11)	29.90 (5.05)	22.30 (5.32)	**<0.001 (0.59)**	7.30 (13.34)	**0.023 (0.41)**
N1 (%)	7.17 (1.31)	7.16 (2.45)	0.689 (0.08)	7.05 (1.28)	5.25 (1.24)	**<0.001 (0.59)**	1.80 (3.10)	**0.013 (0.42)**
N2 (min)	197 (21.10)	200 (26.20)	0.617 (0.04)	198 (23.20)	197 (31.20)	0.668 (0.06)	4.40 (31.92)	0.345 (0.09)
N2 (%)	46.70 (6.07)	47.90 (6.52)	0.265 (0.11)	46.70 (5.85)	46.40 (7.43)	0.565 (0.06)	1.50 (8.21)	0.259 (0.17)
N3 (min)	88 (37.80)	86.40 (45.60)	0.700 (0.12)	88.70 (40.40)	94.20 (40.10)	0.359 (0.09)	−7.10 (55.33)	0.317 (0.16)
N3 (%)	20.60 (8.27)	20.40 (10.20)	0.684 (0.12)	20.60 (8.88)	21.90 (9.03)	0.304 (0.12)	−1.50 (12.60)	0.391 (0.16)
SE (%)	88.70 (3.58)	87.80 (3.47)	0.057 (0.13)	89 (3.23)	89.50 (3.37)	0.188 (0.09)	−1.20 (2.46)	**0.038 (0.32)**
SOL (min)	15.50 (8.57)	15.20 (6.45)	0.667 (0.001)	15.80 (7.78)	11.40 (4.49)	**<0.001 (0.36)**	4.30 (6.10)	**0.004 (0.31)**
WASO (min)	38.50 (13.80)	42.40 (12.40)	0.055 (0.21)	36.40 (12.00)	38.50 (13.80)	0.128 (0.07)	1.70 (10.87)	0.361 (0.11)

HPLC: high-protein, low-carbohydrate; LPHC: low-protein, high carbohydrate; REM: rapid-eye movement; SE: sleep efficiency; SOL: sleep onset latency; TST: total sleep time; WASO: wake after sleep onset; SD: standard deviation; ES: effect size; significant *p* values are bolded.

**Table 3:** A 24-h ad-libitum energy and macronutrients intake during day 4 in response to HPLC vs. LPHC diets.

	HPLC (n = 24)	LPHC (n = 24)	HPLC vs. LPHC	
	Mean (SD)	Mean (SD)	Delta HPLC-LPHC (SD)	*p*-Value (ES) of Differences
Total EI (kcal)	2878.15 (218.65)	2814.38 (185.48)	63.77 (104.87)	**0.027 (0.16)**
Total CHO (g)	337.43 (22.10)	328.50 (18.01)	8.93 (14.15)	**0.007 (0.19)**
Total Fat (g)	114.25 (10.34)	112.17 (14.11)	2.08 (8.63)	0.290 (0.09)
Total Protein (g)	125.05 (28.51)	122.71 (29.30)	2.34 (16.01)	0.875 (0.06)
Breakfast EI (kcal)	606.29 (97.15)	617.13 (121.36)	−10.83 (75.39)	0.681 (0.03)
Breakfast CHO (g)	86.30 (14.12)	79.19 (15.39)	7.12 (15.73)	**0.023 (0.27)**
Breakfast Fat (g)	17.48 (5.12)	21.98 (7.12)	−4.49 (7.36)	**0.016 (0.31)**
Breakfast protein (g)	25.93 (7.61)	25.65 (8.04)	0.28 (6.82)	0.700 (0.05)
Lunch EI (kcal)	1002.35 (144.21)	1004.34 (155.65)	−1.99 (236.57)	0.989 (0.02)
Lunch CHO (g)	116.32 (16.29)	120.92 (21.93)	−4.60 (29.32)	0.242 (0.15)
Lunch Fat (g)	39.55 (7.09)	36.83 (10.22)	2.72 (13.67)	0.169 (0.15)
Lunch protein (g)	45.28 (11.51)	47.30 (12.39)	−2.02 (9.95)	0.219 (0.10)
Dinner EI (kcal)	1001.04 (116.30)	959.73 (71.56)	41.32 (146.50)	0.432 (0.14)
Dinner CHO (g)	99.05 (13.77)	98.94 (12.42)	0.11 (21.18)	0.988 (0.06)
Dinner Fat (g)	48.37 (7.14)	45.00 (7.52)	3.37 (10.79)	0.170 (0.28)
Dinner protein (g)	42.38 (10.09)	39.76 (9.13)	2.63 (7.77)	0.204 (0.15)
Snacking EI (kcal)	268.47 (59.69)	233.19 (80.74)	35.28 (94.34)	**0.002 (0.38)**
Snacking CHO (g)	35.75 (11.88)	29.45 (10.66)	6.30 (13.52)	**0.027 (0.24)**
Snacking Fat (g)	8.85 (5.16)	8.38 (4.25)	0.47 (6.22)	0.944 (0.003)
Snacking protein (g)	11.46 (8.02)	10.00 (7.09)	1.46 (10.38)	0.406 (0.11)

CHO: carbohydrate; EI: energy intake; HPLC: high-protein, low-carbohydrate; LPHC: low-protein, high carbohydrate; significant *p* values are bolded.

**Table 4:** Free living sedentary behaviors and physical activity during day 4 in response to HPLC vs. LPHC diets. HPLC: high-protein, low-carbohydrate; LPA: light physical activity; LPHC: low-protein, high carbohydrate; MPA: moderate physical activity; MVPA: moderate to vigorous physical activity; SB: sedentary behavior; VPA: vigorous physical activity.

	HPLC (n = 24)	LPHC (n = 24)	HPLC vs. LPHC	
	Mean (SD)	Mean (SD)	Delta HPLC-LPHC (SD)	*p*-Value (ES) of Differences
Time spent on SB (min)	485 (30)	482 (24)	3 (20)	0.533 (0.06)
Time spent on LPA (min)	436 (41)	437 (32)	−1 (20)	0.553 (0.05)
Time spent on MPA (min)	35 (16)	40 (17)	−5 (15)	0.117 (0.13)
Time spent on VPA (min)	7 (6)	8 (5)	−1 (5)	0.637 (0.10)
Time spent on MVPA (min)	42 (19)	48 (20)	−6 (15)	0.074 (0.16)

## Supplementary Materials

The following supporting information can be downloaded at: https://www.mdpi.com/article/10.3390/nu14245299/s1, Figure S1. Individual response of REM latency and arousals to HPLC and LPHC diets; Figure S2. Correlation between Δ cortical arousals and Δ CHO; Table S1. Macronutrients content and proportions of HPLC and LPHC diets; Table S2. Food items included in HPLC and LPHC diets; Table S3. Sedentary behaviors and physical activity during HPLC vs. LPHC (Day 1–Day 3); Table S4. Comparison of sleep outcomes during PRE-intervention night between HPLC and LPHC.
