# Supplementary material for: Isocaloric Diets with Different Protein-Carbohydrate Ratios: The Effect on Sleep, Melatonin Secretion and Subsequent Nutritional Response in Healthy Young Men
Source: Nutrients. 2022 Dec 13;14(24):5299. doi: 10.3390/nu14245299 (PMC9782994; doi:10.3390/nu14245299)
Supplement: Supplementary file 1 [file nutrients-14-05299-s001.zip › nutrients-2070917-supplementary.pdf]

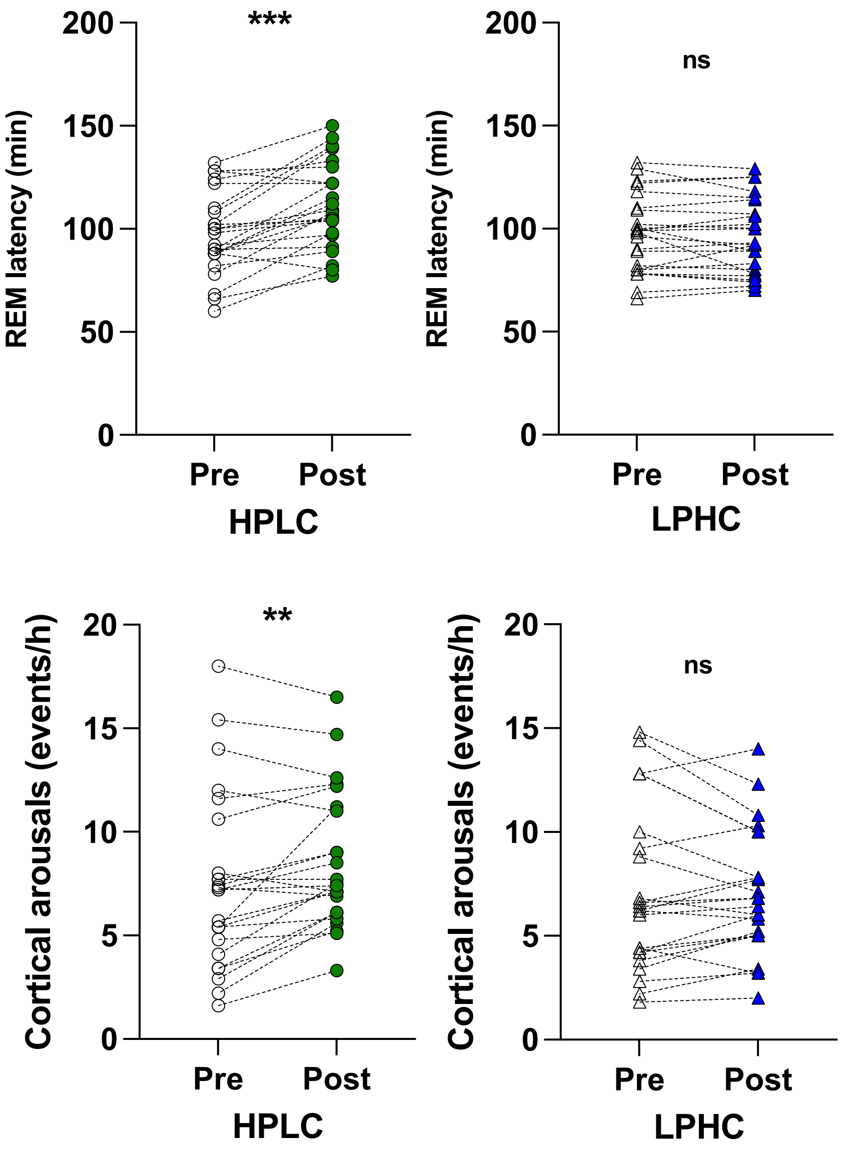

Figure S1. Individual response of REM latency and arousals to HPLC and LPHC diets

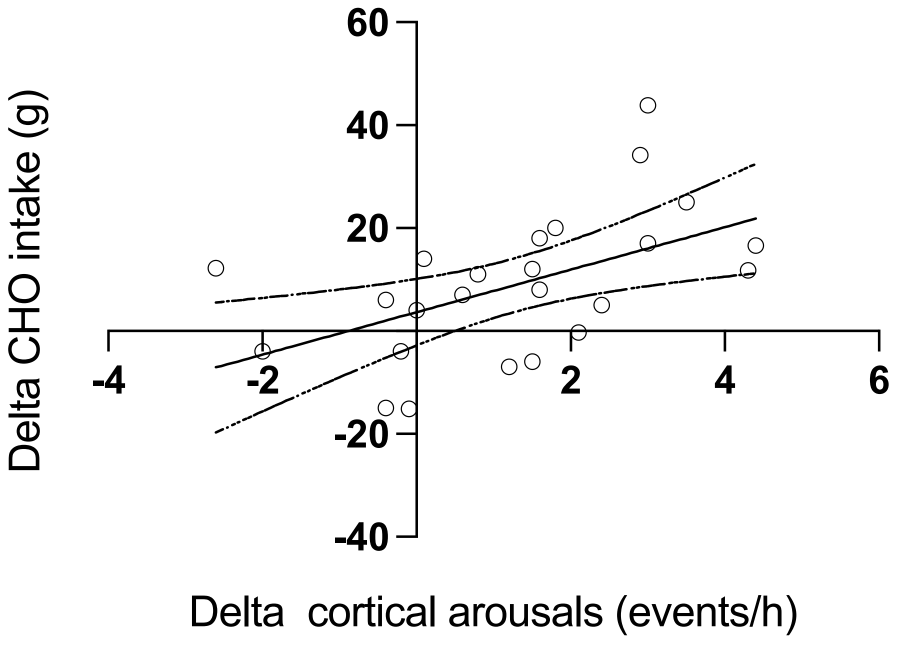

Figure S2. Correlation between Δ cortical arousals and Δ CHO
* (r=0.523, p<0.009); Δ is expressed as (HPLC-LPHC)

Table S1. Macronutrients content and proportions of HPLC and LPHC diets

	HPLC diet	LPHC diet
**Energy (kcal/kg)**	42	42
**Protein (g/kg)**	2	1.1
**Protein (%)**	20	11
**Carbohydrates (g/kg)**	4.45	5.35
**Carbohydrates (%)**	42	51
**Fat (g/kg)**	1.8	1.8
**Fat (%)**	38	38
**Protein : Carbohydrates ratio**	0.44	0.20

HPLC: high-protein, low-carbohydrate; LPHC: low-protein, high carbohydrate

Table S2. Food items included in HPLC and LPHC diets

	Groups	Food	Nutritional values per 100g of food
Breakfast	Dairy products	Milk<br>Yogurt	116 kcal<br>78 kcal
	Fruits	Seasonal fruit (Orange or apple or pear)	67 kcal
	Grain (cereal)	Bread<br>Cereal grains	210 kcal<br>231 kcal
	Sweet product, sugars	Apricot jam	26 kcal
	Lean meats and poultry	Sliced turkey	121 kcal
	Fats	Butter	74 kcal
Lunch	Vegetables and legumes (raw)	Grated carrot	32 kcal
	Fish	White fish fillet	154 kcal
	Grain (cereal)	Steamed rice	133 kcal
	Vegetables and legumes/beans (cooked)	Mixed vegetables (onion, carrot, red and yellow bell pepper)	117 kcal
	Dairy products	Unflavoured yoghurt	62 kcal
	Sweet product, sugars	Applesauce<br>Sugars (5g)	90 kcal<br>398 kcal
Dinner	Vegetables and legumes (raw)	Coleslaw	26 kcal
	Lean meats and poultry	Sliced turkey	121 kcal
	Grain (cereal)	Cooked pasta<br>bread	145 kcal<br>263 kcal
	Vegetables and legumes/beans (cooked)	French bean	27 kcal
	Dairy products	Cottage cheese	74 kcal
	Fruits	Seasonal fruit (Orange or apple or pear)	48 kcal

Table S3. Sedentary behaviors and physical activity during HPLC vs. LPHC diets (Day1-Day3)

	HPLC				LPHC			
	Mean (SD)Day 1	Mean (SD) Day 2	Mean (SD) Day 3	p-value (ES)	Mean (SD) Day 1	Mean (SD) Day 2	Mean (SD) Day 3	p-value (ES)
**Time spent on SED**	489.38 (35.77)	490.38 (29.57)	490.15 (31.42)	0.949 (<0.001)	487.33 (19.10)	487.83 (18.33)	488.29 (29.52)	0.936 (<0.001)
**Time spent on LPA**	427.75 (35.12)	427.63 (35.28)	427.98 (39.85)	0.992 (<0.001)	429.67 (41.59)	429.79 (36.99)	4329.96 (40.43)	0.993 (<0.001)
**Time spent on MPA**	39 (18.44)	38.96 (18.37)	38.94 (19.86)	0.999 (<0.001)	38.63 (16.82)	38.25 (18.15)	38.31 (17.60)	0.957 (<0.001)
**Time spent on VPA**	6.63 (3.75)	6.54 (4.31)	6.32 (4.77)	0.860 (<0.001)	7.42 (4.45)	7.38 (4.83)	7.33 (5.48)	0.995 (<0.001)
**Time spent on MVPA**	45.63 (20.97)	45.50 (21.34)	45.26 (23.20)	0.972 (<0.001)	46.04 (17.64)	45.63 (19.17)	45.64 (19.48)	0.976 (<0.001)

HPLC: high-protein, low-carbohydrate; LPA: light physical activity; LPHC: low-protein, high carbohydrate; MPA: moderate physical activity; MVPA: moderate to vigorous physical activity; SB: sedentary behavior; VPA: vigorous physical activity.

Table S4. Comparison of sleep outcomes during PRE-intervention night between HPLC and LPHC diets

	HPLC	LPHC	p-value (ES)
	Mean (SD)	Mean (SD)	
**TST (min)**	423 (24.00)	426 (24.60)	0.386 (0.05)
**REM (min)**	108 (21.60)	109 (22.10)	0.458 (0.03)
**REM (%)**	25.60 (4.94)	25.70 (5.43)	0.790 (0.01)
**N1 (min)**	30.3 (5.33)	29.90 (5.05)	0.773 (0.03)
**N1 (%)**	7.17 (1.31)	7.05 (1.28)	0.406 (0.06)
**N2 (min)**	197 (21.10)	198 (23.20)	0.473 (0.06)
**N2 (%)**	46.70 (6.07)	46.70 (5.85)	0.856 (0.01)
**N3 (min)**	88 (37.80)	88.70 (40.40)	0.747 (0.001)
**N3 (%)**	20.60 (8.27)	20.60 (8.88)	0.922 (0.02)
**SE (%)**	88.70 (3.58)	89 (3.23)	0.197 (0.03)
**SOL (min)**	15.50 (8.57)	15.80 (7.78)	0.567 (0.06)
**WASO (min)**	38.50 (13.80)	36.40 (12.00)	0.128 (0.07)
**REM Latency (min)**	97.20 (19.90)	97 (18.80)	0.886 (0.02)
**Cortical arousals (events/h)**	7.15 (3.94)	7.43 (4.25)	0.303 (0.03)

HPLC: high-protein, low-carbohydrate; LPHC: low-protein, high carbohydrate; REM: rapid-eye movement; SE: sleep efficiency; SOL: sleep onset latency; TST: total sleep time; WASO: wake after sleep onset
